# The Utility of Nicotinamide N-Methyltransferase as a Potential Biomarker to Predict the Oncological Outcomes for Urological Cancers: An Update

**DOI:** 10.3390/biom11081214

**Published:** 2021-08-16

**Authors:** Roberto Campagna, Valentina Pozzi, Graziana Spinelli, Davide Sartini, Giulio Milanese, Andrea Benedetto Galosi, Monica Emanuelli

**Affiliations:** 1 Department of Clinical Sciences, Polytechnic University of Marche, 60126 Ancona, Italy; rob_campagna@yahoo.com (R.C.); valentinapozzi81@gmail.com (V.P.); grazianaspin@hotmail.it (G.S.); g.milanese@univpm.it (G.M.); a.b.galosi@univpm.it (A.B.G.); m.emanuelli@univpm.it (M.E.); 2 New York-Marche Structural Biology Center (NY-MaSBiC), Polytechnic University of Marche, 60131 Ancona, Italy

**Keywords:** nicotinamide N-methyltransferase, renal cell carcinoma, bladder cancer, prostate cancer, tumor biomarker, molecular target

## Abstract

Nicotinamide N-methyltransferase (NNMT) catalyzes the N-methylation reaction of nicotinamide, using S-adenosyl-L-methionine as the methyl donor. Enzyme overexpression has been described in many non-neoplastic diseases, as well as in a wide range of solid malignancies. This review aims to report and discuss evidence available in scientific literature, dealing with NNMT expression and the potential involvement in main urologic neoplasms, namely, renal, bladder and prostate cancers. Data illustrated in the cited studies clearly demonstrated NNMT upregulation (pathological vs. normal tissue) in association with these aforementioned tumors. In addition to this, enzyme levels were also found to correlate with key prognostic parameters and patient survival. Interestingly, NNMT overexpression also emerged in peripheral body fluids, such as blood and urine, thus leading to candidate the enzyme as promising biomarker for the early and non-invasive detection of these cancers. Examined results undoubtedly showed NNMT as having the capacity to promote cell proliferation, migration and invasiveness, as well as its potential participation in fundamental events highlighting cancer progression, metastasis and resistance to chemo- and radiotherapy. In the light of this evidence, it is reasonable to attribute to NNMT a promising role as a potential biomarker for the diagnosis and prognosis of urologic neoplasms, as well as a molecular target for effective anti-cancer treatment.

## 1. Introduction

**Figure 1:**
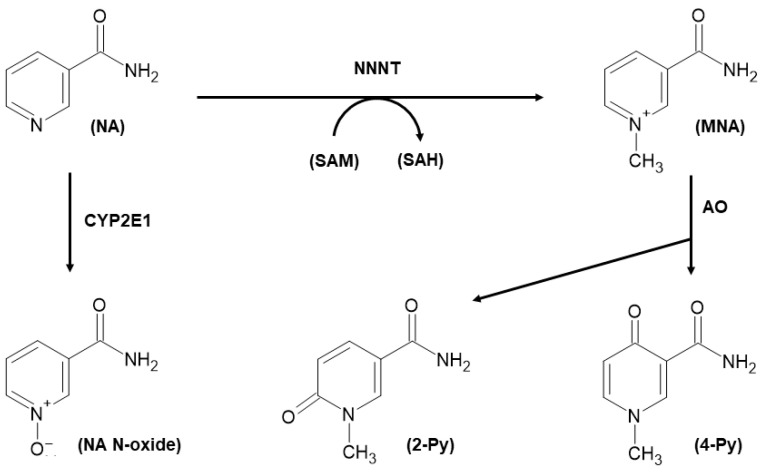
Nicotinamide (NA) catabolism through N-methylation and N-oxidation. NA is methylated by nicotinamide N-methyltransferase (NNMT), using the universal methyl donor S-adenosyl-L-methionine (SAM) that is converted to S-adenosyl-L-homocysteine (SAH). The methylated product N1-methylnicotinamide (MNA) can be further oxidized into N1-methyl-2-pyridone-5-carboxamide (2-Py), or N1-methyl-4-pyridone-3-carboxamide (4-Py), by aldehyde oxidase (AO). NA can be oxidized to nicotinamide N-oxide (NA N-oxide) by CYP2E1 enzyme. N-methylated and N-oxidized metabolites are excreted through urine.

The enzyme nicotinamide N-methyltransferase (NNMT, EC 2.1.1.1) catalyzes the transfer of the methyl group from the S-adenosyl-L-methionine (SAM) universal donor to nicotinamide (NA) to form N1-methylnicotinamide (MNA) and S-adenosyl-L-homocysteine (SAH) [1,2,3]. MNA can be further converted into N1-methyl-2-pyridone-5-carboxamide (2-Py) or N1-methyl-4-pyridone-3-carboxamide (4-Py), through the catalysis exerted by aldehyde oxidase (AO) [4]. All three catabolites (NA, 2-Py and 4-Py) are subsequently excreted via urine [5]. Alternatively, NA excess can be also metabolized through oxidation rather than methylation. Indeed, within the endoplasmic reticulum of hepatocytes, the CYP2E1 enzyme system is able to oxidize NA to nicotinamide N-oxide (NA N-oxide) that undergoes further urinary elimination [6] (Figure 1).

NA represents a bioactive form of vitamin B3, and is the precursor of nicotinamide adenine dinucleotide (NAD^+^), a key molecule participating in fundamental redox processes regulating energy metabolism [7], as well as to a wide range of intracellular events, including transcription regulation, longevity, genome stability, and response to DNA damage [8]. NNMT catalytic activity significantly contributes to the regulation of NA intracellular levels, participating to an irreversible step of catabolism of vitamin B3, that undergoes excretion after N-methylation. Upon hepatic conversion to MNA and further pyridones, NA is excreted through urine and is no longer available as a precursor for NAD^+^ biosynthesis [9].

NNMT is mainly expressed in the liver and belongs to phase II metabolizing enzymes [1]. Although the enzyme is described to be involved in the biotransformation and detoxification of many xenobiotics [1,2,3], porcine liver NNMT was only described to be active towards pyridine derivatives, such as quinoline, isoquinoline, and 1,2,3,4-tetrahydroisoquinoline, in addition to structural NA analogs, such as thionicotinamide and 3-acetylpyridine [10]. The resolution of tridimensional (3D) structures of purified human recombinant NNMT, together with the detection of the main amino acid residues involved in the catalysis [11], led to the discovery of the kinetic mechanism of NNMT [12], the identification of alternate enzyme substrates [13,14,15], as well as the design and assay of a great number of inhibitors [16,17,18,19,20,21,22,23,24,25,26,27].

NNMT upregulation, as well as its involvement, has been described for several non-neoplastic disorders, such as Parkinson’s disease [28,29,30,31], metabolic disfunctions [32,33,34,35], chronic obstructive pulmonary disease [36,37], atherosclerosis [38] and pulmonary arterial hypertension [39].

However, the bulk of scientific literature on NNMT is greatly focused on speculating and clarifying the role played by the enzyme in cancer. In fact, NNMT overexpression has been reported for many solid tumors, including gastrointestinal neoplasms [40,41,42,43,44,45,46,47,48], lung [49,50,51,52], oral [53,54,55,56,57,58,59], esophageal [60,61], nasopharyngeal [62] and thyroid [63,64,65] cancers, as well as ameloblastoma [66] and glioblastoma multiforme [67]. Significant NNMT upregulation was recently found in epithelial neoplasms [68,69,70,71,72,73] and in association with cancer stem cells (CSCs) [74,75,76,77,78]. Regarding women’s cancers, enzyme overexpression was detected in breast [79,80,81], endometrial [82], cervical [83] and ovarian [84] cancer.

The aim of this review was to give an update on data concerning NNMT expression levels in urologic malignancies, such as renal, bladder and prostate cancer, as well as to report evidence showing enzyme contributions in malignant transformations featuring these neoplasms, and the role played by NNMT in tumor cell metabolism and phenotypes.

## 2. NNMT and Renal Cancer

Among urological neoplasms, renal cell carcinoma (RCC) was the first to be examined in regard to potential NNMT involvement, in which enzyme overexpression was described.

In 2013, RCC was reported to rank seventh among all cancers worldwide. It was diagnosed in more than 350,000 people and was responsible for 140,000 deaths [85]. RCC represents a heterogenous group of neoplasms, including different histological subtypes. Conventional clear cell (ccRCC), papillary (pRCC) and chromophobe (chRCC) are the main tumor forms, accounting for 85–90% of all kidney malignancies [86]. Cancer management and patient survival are strictly dependent on the disease stage at the time of diagnosis. Surgery, followed by active surveillance, remains the gold standard curative intervention for localized tumors, whereas systemic immunotherapy is adopted to treat metastatic disease [87]. However, the prognosis of patients with metastases is poor, mainly due to the lack of effective chemotherapy [88]. In the light of these considerations, improvement in the understanding of RCC biology, focused on identifying new molecular targets for effective therapy, is of utmost importance.

In the work by Yao et al., microarrays and Real-Time PCR analyses were used to identify molecular biomarkers for RCC diagnosis, distinguishing between ccRCC, pRCC and chRCC subtypes, as well as for prognostic purpose. Results obtained revealed that NNMT upregulation (tumor vs. normal tissue) was markedly higher in ccRCC than in other non-clear cell RCCs. Interestingly, enzyme levels were significantly higher in small-sized rather than large-sized tumors [89].

This evidence was further confirmed by our study, which aimed to explore the involvement of phase II drug metabolism enzymes in RCC. Differential gene expression measurements, performed by Real-Time PCR, Western blot, and catalytic activity assay, demonstrated that NNMT levels significantly increase in tumors compared with normal kidney samples of patients affected with ccRCC. On the contrary, enzyme expression in neoplastic was not so altered with respect to the control specimens of subjects suffering from chRCC, as well as oncocytoma. Similarly to what was previously reported, NNMT expression was found to be inversely related to tumor size [90].

Immunohistochemical analyses were performed to deeply explore the potential use of NNMT level determination for differential diagnosis, as well as the prognosis prediction of renal cancer. Data reported by the study of Zhang et al. clearly demonstrated higher enzyme positivity in ccRCC in comparison with the chromophobe subtype. Moreover, NNMT expression was significantly inversely related to the status of pT parameter. Univariate survival analysis revealed that elevated enzyme levels were associated with a poor prognosis. Indeed, Kaplan–Meier curve trends displayed a longer survival time for patients affected with tumors exhibiting low NNMT expression, and vice versa [91].

In the study by Kim et al., two-dimensional polyacrylamide gel electrophoresis (2D-PAGE) and mass spectrometry (MS) were used to analyze frozen tumor and adjacent normal tissue samples from patients affected by ccRCC, pRCC and chRCC. Further validation was carried out on formalin-fixed and paraffin-embedded tissue specimens, as well as on plasma samples, through immunohistochemistry and ELISA, respectively. Data obtained revealed that NNMT was upregulated in most of the analyzed samples, representing, therefore, a powerful potential biological marker for the diagnosis of the main RCC subtypes [92]. Subsequent determination of NNMT, L-plastin (LCP1), and nonmetastatic cells 1 protein (NM23A) levels, performed in a wide number of plasma samples obtained from healthy donors and RCC patients, was used to explore the clinical performance of a three-marker assay for the early diagnosis of kidney cancer. The resulting area under curve (AUC), as well as sensitivity and specificity values, obtained from receiver operating characteristic (ROC) analysis, demonstrated the elevated diagnostic accuracy derived from the composite three-marker assay for early RCC detection [93,94].

Further proteomic approaches were used to explore the potential suitability of NNMT expression level determination for a valuable diagnosis of RCC, focusing on conventional subtypes. Reported data showed that, in addition to a few other genes, NNMT was identified as a reliable biomarker for ccRCC detection [95], especially for late-stage disease [96].

Although several studies described NNMT upregulation in RCC, the molecular events leading to enzyme overexpression, as well as the effect induced by such dysregulation in cancer cell phenotype, remain partly understood. In the last decade, a few studies have been carried out in order to elucidate these aspects.

We recently reported data from Real-Time PCR analyses, performed on paired ccRCC and controlled tissue samples, of molecules potentially involved in the modulation of NNMT expression at the transcriptional level. In particular, we focused on signal transducers and activators of transcription 3 (STAT3), interleukin 6 (IL-6) and hepatocyte nuclear factor 1 beta (HNF-1β) transcription factors, as well as on transforming growth factor beta 1 (TGF-β1) cytokines. The obtained results clearly demonstrated that, among these transcriptional regulators, TGF-β1 only displayed a significant upregulation in tumor compared with normal tissue samples of ccRCC patients [97].

Most of the studies devoted to elucidating NNMT contributions to the malignant transformation of kidney cells, by promoting proliferation, migration, invasiveness and resistance to therapy, had been carried out on primary (769-P and 786-O) and metastatic (Caki-1) ccRCC cell lines, as well as in primary (Caki-2) pRCC cells.

Preliminary results, reported by Kim et al., illustrated that short hairpin RNA (shRNA)-mediated NNMT silencing was associated with a significant decrease in proliferation of Caki-1 cells [92]. Treatment with small interfering RNAs (siRNAs) targeting NNMT efficiently suppressed the invasive capacity of 786-O and Caki-1 cells, whereas the induction of enzyme overexpression led to an opposite effect in both 769-P and human embryonic kidney cell line HEK293. Due to the discovery of a strong correlation between expression levels of NNMT and matrix metalloproteinase-2 (MMP-2), that is suggested to play an important role in cancer cell invasion, further analyses were carried out to disclose the interplay between both determinants of cellular invasiveness. siRNA-mediated NNMT knockdown efficiently decreased MPP-2 expression in 786-O and Caki-1 cells. Moreover, MPP-2 suppression, via a neutralizing antibody or inhibitor, significantly inhibited the invasive capacity of HEK293 cells overexpressing NNMT, suggesting that NNMT-dependent cellular invasion was exerted through the activation of MMP-2. In particular, MMP-2 transcriptional activity, induced by NNMT upregulation in HEK293, was found to be strictly regulated by SP1-binding elements within the promoter region. Further analyses, performed on HEK293 and ccRCC cell lines, revealed that SP1 binding capacity to MMP-2 promoter was positively regulated by activation of the PI3K-Akt signaling pathway. In vivo studies demonstrated that tumor growth and lung metastasis were significantly reduced in immunodeficient mice subcutaneously and intravenously injected with 786-O downregulating NNMT [98].

Caki-1 treatment with a sulfonamide analogue of alkaloid cryptopleurine led to a significant reduction in proliferation and cell cycle progression, as well as to NNMT downregulation. Interestingly, antiproliferative activity and cell cycle arrest exerted by this pharmacological treatment were inhibited or abolished upon siRNA-mediated NNMT silencing. Further analyses demonstrated that NNMT knockdown was associated with decreased levels of phosphorylated c-Jun, whose accumulation is responsible for G0/G1 cell cycle arrest. This evidence seems to suggest a potential involvement of NNMT in molecular events connected with the antiproliferative and cell cycle-regulating effects induced by Caki-1 cell treatment with a methanesulfonamide analogue of cryptopleurine [99].

## 3. NNMT and Bladder Cancer

In the context of studies aiming to disclose the potential involvement of NNMT in urothelial neoplasms, array analyses were used to profile the expression of tumor and normal-looking tissue samples obtained from patients affected with bladder cancer (BC) [100,101].

BC is the fourth most common neoplasm occurring in men in Western countries, after prostate, lung and colon cancers. It is a heterogeneous disease and 90–95% cases are represented by urothelial carcinoma or transitional cell carcinoma. BC is three to four times more frequent in men than in women, and smoking habits, together with occupational activity, constitute the main environmental risk factors [102,103]. At the time of diagnosis, 60% of BC patients suffer from low-grade, non-muscle invasive forms (NMIBC), whereas 25% are affected by muscle invasive disease (MIBC), displaying high histological grade and being responsible for the vast majority of cancer-related deaths. After the transurethral endoscopic resection of a bladder tumor, often followed by chemotherapy or vaccine-based treatment, most NMIBC patients undergo cancer relapses, 10–20% of which are represented by high-grade MIBCs. Due to cancer recurrence and/or progression, BC patients are monitored through periodic cystoscopies and urine cytology examinations. Cystoscopy is an invasive and expensive procedure, often leading to pain for patients. Urine cytology displays low sensitivity in detecting low-grade tumors. NMIBC patients harboring high-grade neoplasms undergo major surgery for bladder removal, through partial or radical cystectomy. Chemotherapy and radiotherapy are adopted in combination with surgery for treating patients affected by MIBC, or are used alone to manage metastatic disease [104,105]. Therefore, the identification of sensitive and non-invasive biomarkers for BC detection and follow-up, as well as molecular targets for effective treatment of cancer progression, are urgently required.

Among enzymes involved in drug metabolism, NNMT was found to be significantly overexpressed in BC, evidence that was further confirmed by Real-Time PCR, Western blot and catalytic activity assay [100]. Subsequent investigation of differentially expressed genes in relation to the BC stage revealed that NNMT, together with fibronectin 1 (FN1), POSTN and SMAD6, were markedly upregulated in muscle-invasive tumors compared with non-muscle-invasive tumors [101]. Results obtained from these distinct studies reasonably suggest NNMT as a potential biomarker for both BC diagnosis and prognosis.

NNMT levels were also detected in exfoliated cells collected from urine samples obtained from BC patients and healthy subjects. The data reported showed that enzyme expression was significantly higher in the urine of patients suffering from BC compared with that detected in control specimens. Subsequent statistical analyses revealed a significant inverse correlation between tumor NNMT levels and histological grade. Moreover, ROC analysis, as well as AUC evaluation, demonstrated the excellent diagnostic accuracy of a potential urine-based NNMT test. Such evidence strongly indicates that NNMT level determination could be used for early and non-invasive BC detection [106].

The pivotal study, that started to elucidate the role of NNMT in bladder cancer cells, used the cDNA array technique to profile the expression of stress-related and DNA repair genes in both radioresistant MGH-U1 BC cell line and its radiosensitive subclone S40b. Among dysregulated genes, the NNMT displayed marked decreased levels in S40b with respect to those detected in MGH-U1 cells. This result, further confirmed by Real-Time PCR assay, represented the first evidence suggesting a potential role played by the enzyme in cell responses to radiation treatment [107].

Wu et al. were the first to report NNMT involvement in BC cell metabolism, focusing on events featuring cell migration. The goal of this in vitro study was to detect genes promoting invasion and metastasis. To this aim, numerous human BC cell lines were both subjected to radial migration assay and then microarray-profiled. NNMT was identified among genes whose expression was directly correlated with cell migration rate, and therefore selected for further investigation. Data obtained from analyses carried out in BC tissue samples revealed that enzyme expression was also positively associated with the tumor stage, and was found to be higher in muscle-invasive forms compared with that of superficial neoplasms. Given these results, further in vitro experiments were performed in order to speculate the potential mechanistic role of NNMT in cell migration. Interestingly, siRNA-mediated enzyme downregulation significantly reduced cell proliferation, as well as chemotaxis and chemokinesis in the 253J human BC cell line [108].

## 4. NNMT and Prostate Cancer

It is only recently that NNMT was found to be upregulated in prostate cancer (PCa). PCa represents a major disease that affects men’s health and is the second most common tumor in men worldwide, being a leading cause of cancer morbidity and mortality in developed countries [85]. PCa is a complex and heterogeneous neoplasm whose diagnosis is currently based on digital rectal examination, prostate-specific anti-gene measurement and transrectal ultrasound. Among prognostic parameters, histological grading assigned according to Gleason score is the strongest predictive factor of clinical outcome and response to treatment [109]. Active surveillance is adopted for low-risk patients, whereas subjects diagnosed with high-risk disease are treated with radical prostatectomy or radiotherapy [110]. Since androgens are essential for the development of the prostate gland, but also stimulate prostate cancer cell growth, patients affected by locally advanced PCa are treated with androgen-deprivation therapy (ADT), combined with radiotherapy, in order to prevent tumor progression. However, over a period of 12–36 months, patients develop castrate-resistant prostate cancer (CRPC), as a result of the activation of the androgen receptor and its downstream signaling pathway. Chemotherapy is used in combination with ADT in patients with distant metastasis or administered in subjects affected by metastatic CRPC [109,110]. Therefore, it is fundamental to identify and validate new tumor-associated biomarkers, in order to improve the management of PCa patients, in terms of cancer detection, follow-up and therapy.

In the study of Zhou et al., immunohistochemistry was carried out to explore enzyme expression in benign prostate hyperplasia (BPH), high-grade prostatic intraepithelial neoplasia (HGPIN) and PCa. The results obtained showed that NNMT levels were significantly higher in HGPIN or PCa compared with those of BPH. Subsequent statistical analyses revealed an inverse correlation between intratumor enzyme expression and Gleason score. Moreover, low NNMT levels were found to be associated with both reduced progression-free survival and overall survival of patients affected by advanced PCa [111]. Enzyme expression was also investigated at mRNA level in peripheral blood samples of PCa patients and healthy subjects. The reported data clearly demonstrated that NNMT was upregulated in pathological specimens compared with controls [112]. This evidence led to hypotheses that NNMT could serve as a promising biomarker for early and non-invasive diagnosis, as well as the prognosis of prostate cancer [111,112].

In order to speculate the role played by the enzyme in cancer cell phenotype, further analyses were carried out in the PC-3 prostate adenocarcinoma cell line. The induction of NNMT upregulation was associated with a significant increase in cell viability and migration, as well as colony formation capacity. On the contrary, siRNA-mediated enzyme downregulation led to a reduced proliferation of PC-3 cells. Interestingly, NNMT overexpression also enhanced sirtuin 1 (SIRT1) levels, suggesting a potential involvement of this histone deacetylase among mechanisms through which NNMT is able to promote PCa malignancy. To verify this hypothesis, PC-3 cells were treated with NA, known to be a strong SIRT1 inhibitor, and subsequent molecular and phenotypic effects were also evaluated. NA treatment significantly reduced SIRT1 expression and cell migration, both in PC-3 cells overexpressing NNMT and controls, thus indicating that the enzyme capacity to promote PC-3 cell malignancy is mediated by SIRT1 [112,113].

## 5. Conclusions

To the best of our knowledge, this is the first review reporting the concerns regarding the role played by NNMT in urological tumors, focusing on enzyme expression levels in tissues and body fluids, as well as its involvement in molecular and cellular events promoting malignant transformation.

First classified as an NA clearance enzyme [114], NNMT has gradually acquired numerous functions as a fundamental molecule able to regulate metabolism and epigenetics, thus affecting many aspects related to cellular physiology and pathology [115].

For almost twenty years, NNMT has been described to be upregulated in several solid cancers, participating in crucial mechanisms leading to cancer progression, metastasis and resistance to chemo- and radiotherapy.

**Figure 2:**
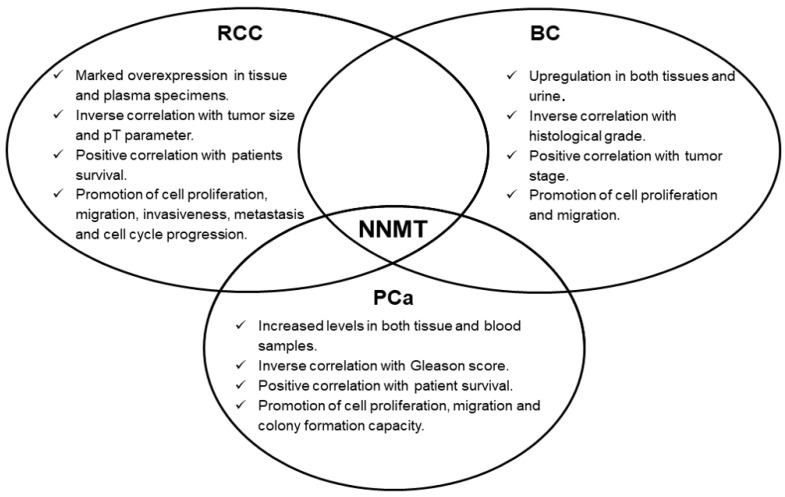
Nicotinamide N-methyltransferae (NNMT) involvement in urological tumors. Enzyme expression (tumor vs. control tissue) and its role played in cellular phenotype were explored in main urinary tract neoplasms, namely renal cell carcinoma (RCC), bladder cancer (BC) and prostate cancer (PCa).

Concerning urologic malignancies, enzyme overexpression emerged in renal, bladder and prostate cancers. In these neoplasms, NNMT seems to act as an interesting diagnostic biomarker, whose measurement might even be suitable for non-invasive and early cancer detection. Moreover, the prognostic potential of NNMT level determination was also demonstrated, with enzyme intratumor expression significantly correlated with important parameters, such as tumor stage, size, grade, as well as with patient survival. Subsequent studies clearly demonstrated NNMT involvement in fundamental events featuring tumorigenesis, namely, cell proliferation, migration and invasiveness (Figure 2), thus attributing to the enzyme a promising role as a molecular target for effective treatment of these tumors. NNMT 3D structure resolution [11], together with the identification of related kinetic mechanism-of-action [12], have triggered the design, synthesis and validation of a wide range of molecules that seem to act as potent inhibitors of enzyme activity [16,17,18,19,20,21,22,23,24,25,26,27], that could be used as novel anti-cancer therapeutics.

In addition to this, recent studies showed that the enrichment of tumor cell lines with CSCs was significantly associated with the enhanced expression of NNMT [76,78]. Acquisition of stem-like phenotype by cancer cells was demonstrated to have detrimental effects in terms of tumor aggressiveness and its resistance to therapy, thus leading to the relapse of neoplasm and metastatic spread [116]. In the light of these considerations and based on data reported in this review, it is conceivable to hypothesize NNMT involvement in those molecular processes and cellular events featuring poor clinical outcomes of urological malignancies. Once again, targeting NNMT could represent a powerful molecular strategy to effectively treat cancer.

## Data Availability

Not applicable.

## References

[1] Rini J., Szumlanski C., Guerciolini R., Weinshilboum R.M. (1990). Human liver nicotinamide N-methyltransferase: Ion-pairing radiochemical assay, biochemical properties and individual variation. Clin. Chim. Acta.

[2] Aksoy S., Szumlanski C.L., Weinshilboum R.M. (1994). Human liver nicotinamide N-methyltransferase. cDNA cloning, expression, and biochemical characterization. J. Biol. Chem..

[3] Aksoy S., Brandriff B.F., Ward A., Little P.F., Weinshilboum R.M. (1995). Human nicotinamide N-methyltransferase gene: Molecular cloning, structural characterization and chromosomal localization. Genomics.

[4] Felsted R.L., Chaykin S. (1967). N1-methylnicotinamide oxidation in a number of mammals. J. Biol. Chem..

[5] Okamoto H., Ishikawa A., Yoshitake Y., Kodama N., Nishimuta M., Fukuwatari T., Shibata K. (2003). Diurnal variations in human urinary excretion of nicotinamide catabolites: Effects of stress on the metabolism of nicotinamide. Am. J. Clin. Nutr..

[6] Real A.M., Hong S., Pissios P. (2013). Nicotinamide N-oxidation by CYP2E1 in human liver microsomes. Drug Metab. Dispos..

[7] Makarov M.V., Trammell S.A.J., Migaud M.E. (2019). The chemistry of the vitamin B3 metabolome. Biochem. Soc. Trans..

[8] Zhang J. (2003). Are poly(ADP-ribosyl)ation by PARP-1 and deacetylation by Sir2 linked?. Bioessays.

[9] Brachs S., Polack J., Brachs M., Jahn-Hofmann K., Elvert R., Pfenninger A., Bärenz F., Margerie D., Mai K., Spranger J. (2019). Genetic Nicotinamide N-Methyltransferase (Nnmt) Deficiency in Male Mice Improves Insulin Sensitivity in Diet-Induced Obesity but Does Not Affect Glucose Tolerance. Diabetes.

[10] Alston T.A., Abeles R.H. (1988). Substrate specificity of nicotinamide methyltransferase isolated from porcine liver. Arch. Biochem. Biophys..

[11] Peng Y., Sartini D., Pozzi V., Wilk D., Emanuelli M., Yee V.C. (2011). Structural basis of substrate recognition in human nicotinamide N-methyltransferase. Biochemistry.

[12] Loring H.S., Thompson P.R. (2018). Kinetic Mechanism of Nicotinamide N-Methyltransferase. Biochemistry.

[13] van Haren M.J., Sastre Toraño J., Sartini D., Emanuelli M., Parsons R.B., Martin N.I. (2016). A Rapid and Efficient Assay for the Characterization of Substrates and Inhibitors of Nicotinamide N-Methyltransferase. Biochemistry.

[14] Thomas M.G., Sartini D., Emanuelli M., van Haren M.J., Martin N.I., Mountford D.M., Barlow D.J., Klamt F., Ramsden D.B., Reza M. (2016). Nicotinamide N-methyltransferase catalyses the N-methylation of the endogenous β-carboline norharman: Evidence for a novel detoxification pathway. Biochem. J..

[15] van Haren M.J., Thomas M.G., Sartini D., Barlow D.J., Ramsden D.B., Emanuelli M., Klamt F., Martin N.I., Parsons R.B. (2018). The kinetic analysis of the N-methylation of 4-phenylpyridine by nicotinamide N-methyltransferase: Evidence for a novel mechanism of substrate inhibition. Int. J. Biochem. Cell. Biol..

[16] Neelakantan H., Wang H.Y., Vance V., Hommel J.D., McHardy S.F., Watowich S.J. (2017). Structure-Activity Relationship for Small Molecule Inhibitors of Nicotinamide N-Methyltransferase. J. Med. Chem..

[17] van Haren M.J., Taig R., Kuppens J., Sastre Toraño J., Moret E.E., Parsons R.B., Sartini D., Emanuelli M., Martin N.I. (2017). Inhibitors of nicotinamide N-methyltransferase designed to mimic the methylation reaction transition state. Org. Biomol. Chem..

[18] Neelakantan H., Vance V., Wetzel M.D., Wang H.L., McHardy S.F., Finnerty C.C., Hommel J.D., Watowich S.J. (2018). Selective and membrane-permeable small molecule inhibitors of nicotinamide N-methyltransferase reverse high fat diet-induced obesity in mice. Biochem. Pharmacol..

[19] Kannt A., Rajagopal S., Kadnur S.V., Suresh J., Bhamidipati R.K., Swaminathan S., Hallur M.S., Kristam R., Elvert R., Czech J. (2018). A small molecule inhibitor of Nicotinamide N-methyltransferase for the treatment of metabolic disorders. Sci. Rep..

[20] Ruf S., Hallur M.S., Anchan N.K., Swamy I.N., Murugesan K.R., Sarkar S., Narasimhulu L.K., Putta V.P.R.K., Shaik S., Chandrasekar D.V. (2018). Novel nicotinamide analog as inhibitor of nicotinamide N-methyltransferase. Bioorg. Med. Chem. Lett..

[21] Lee H.Y., Suciu R.M., Horning B.D., Vinogradova E.V., Ulanovskaya O.A., Cravatt B.F. (2018). Covalent inhibitors of nicotinamide N-methyltransferase (NNMT) provide evidence for target engagement challenges in situ. Bioorg. Med. Chem. Lett..

[22] Neelakantan H., Brightwell C.R., Graber T.G., Maroto R., Wang H.L., McHardy S.F., Papaconstantinou J., Fry C.S., Watowich S.J. (2019). Small molecule nicotinamide N-methyltransferase inhibitor activates senescent muscle stem cells and improves regenerative capacity of aged skeletal muscle. Biochem. Pharmacol..

[23] Gao Y., van Haren M.J., Moret E.E., Rood J.J.M., Sartini D., Salvucci A., Emanuelli M., Craveur P., Babault N., Jin J. (2019). Bisubstrate Inhibitors of Nicotinamide N-Methyltransferase (NNMT) with Enhanced Activity. J. Med. Chem..

[24] Policarpo R.L., Decultot L., May E., Kuzmič P., Carlson S., Huang D., Chu V., Wright B.A., Dhakshinamoorthy S., Kannt A. (2019). High-Affinity Alkynyl Bisubstrate Inhibitors of Nicotinamide N-Methyltransferase (NNMT). J. Med. Chem..

[25] Chen D., Li L., Diaz K., Iyamu I.D., Yadav R., Noinaj N., Huang R. (2019). Novel Propargyl-Linked Bisubstrate Analogues as Tight-Binding Inhibitors for Nicotinamide N-Methyltransferase. J. Med. Chem..

[26] Kannt A., Rajagopal S., Hallur M.S., Swamy I., Kristam R., Dhakshinamoorthy S., Czech J., Zech G., Schreuder H., Ruf S. (2021). Novel Inhibitors of Nicotinamide-N-Methyltransferase for the Treatment of Metabolic Disorders. Molecules.

[27] Akar S., Duran T., Azzawri A.A., Koçak N., Çelik Ç., Yıldırım H.İ. (2021). Small molecule inhibitor of nicotinamide N-methyltransferase shows anti-proliferative activity in HeLa cells. J. Obstet. Gynaecol..

[28] Aoyama K., Matsubara K., Kondo M., Murakawa Y., Suno M., Yamashita K., Yamaguchi S., Kobayashi S. (2001). Nicotinamide-N-methyltransferase is higher in the lumbar cerebrospinal fluid of patients with Parkinson’s disease. Neurosci. Lett..

[29] Parsons R.B., Smith M.L., Williams A.C., Waring R.H., Ramsden D.B. (2002). Expression of nicotinamide N-methyltransferase (E.C. 2.1.1.1) in the Parkinsonian brain. J. Neuropathol. Exp. Neurol..

[30] Parsons R.B., Smith S.W., Waring R.H., Williams A.C., Ramsden D.B. (2003). High expression of nicotinamide N-methyltransferase in patients with idiopathic Parkinson’s disease. Neurosci. Lett..

[31] Williams A.C., Ramsden D.B. (2005). Autotoxicity, methylation and a road to the prevention of Parkinson’s disease. J. Clin. Neurosci..

[32] Liu M., Li L., Chu J., Zhu B., Zhang Q., Yin X., Jiang W., Dai G., Ju W., Wang Z. (2015). Serum N(1)-Methylnicotinamide Is Associated With Obesity and Diabetes in Chinese. J. Clin. Endocrinol. Metab..

[33] Kannt A., Pfenninger A., Teichert L., Tönjes A., Dietrich A., Schön M.R., Klöting N., Blüher M. (2015). Association of nicotinamide-N-methyltransferase mRNA expression in human adipose tissue and the plasma concentration of its product, 1-methylnicotinamide, with insulin resistance. Diabetologia.

[34] Giuliante R., Sartini D., Bacchetti T., Rocchetti R., Klöting I., Polidori C., Ferretti G., Emanuelli M. (2015). Potential involvement of nicotinamide N-methyltransferase in the pathogenesis of metabolic syndrome. Metab. Syndr. Relat. Disord..

[35] Ehebauer F., Ghavampour S., Kraus D. (2020). Glucose availability regulates nicotinamide N-methyltransferase expression in adipocytes. Life Sci..

[36] Debigaré R., Maltais F., Côté C.H., Michaud A., Caron M., Mofarrahi M., Leblanc P., Hussain S.N. (2008). Profiling of mRNA expression in quadriceps of patients with COPD and muscle wasting. COPD.

[37] Kim H.C., Mofarrahi M., Vassilakopoulos T., Maltais F., Sigala I., Debigare R., Bellenis I., Hussain S.N. (2010). Expression and functional significance of nicotinamide N-methyl transferase in skeletal muscles of patients with chronic obstructive pulmonary disease. Am. J. Respir. Crit. Care Med..

[38] Mateuszuk Ł., Khomich T.I., Słomińska E., Gajda M., Wójcik L., Łomnicka M., Gwóźdź P., Chłopicki S. (2009). Activation of nicotinamide N-methyltrasferase and increased formation of 1-methylnicotinamide (MNA) in atherosclerosis. Pharmacol. Rep..

[39] Fedorowicz A., Mateuszuk Ł., Kopec G., Skórka T., Kutryb-Zając B., Zakrzewska A., Walczak M., Jakubowski A., Łomnicka M., Słomińska E. (2016). Activation of the nicotinamide N-methyltransferase (NNMT)-1-methylnicotinamide (MNA) pathway in pulmonary hypertension. Respir. Res..

[40] Jang J.S., Cho H.Y., Lee Y.J., Ha W.S., Kim H.W. (2004). The differential proteome profile of stomach cancer: Identification of the biomarker candidates. Oncol. Res..

[41] Lim B.H., Cho B.I., Kim Y.N., Kim J.W., Park S.T., Lee C.W. (2006). Overexpression of nicotinamide N-methyltransferase in gastric cancer tissues and its potential post-translational modification. Exp. Mol. Med..

[42] Chen C., Wang X., Huang X., Yong H., Shen J., Tang Q., Zhu J., Ni J., Feng Z. (2016). Nicotinamide N-methyltransferase: A potential biomarker for worse prognosis in gastric carcinoma. Am. J. Cancer Res..

[43] Roessler M., Rollinger W., Palme S., Hagmann M.L., Berndt P., Engel A.M., Schneidinger B., Pfeffer M., Andres H., Karl J. (2005). Identification of nicotinamide N-methyltransferase as a novel serum tumor marker for colorectal cancer. Clin. Cancer Res..

[44] Xie X., Yu H., Wang Y., Zhou Y., Li G., Ruan Z., Li F., Wang X., Liu H., Zhang J. (2014). Nicotinamide N-methyltransferase enhances the capacity of tumorigenesis associated with the promotion of cell cycle progression in human colorectal cancer cells. Arch. Biochem. Biophys..

[45] Xie X., Liu H., Wang Y., Zhou Y., Yu H., Li G., Ruan Z., Li F., Wang X., Zhang J. (2016). Nicotinamide N-methyltransferase enhances resistance to 5-fluorouracil in colorectal cancer cells through inhibition of the ASK1-p38 MAPK pathway. Oncotarget.

[46] Song M., Li Y., Miao M., Zhang F., Yuan H., Cao F., Chang W., Shi H., Song C. (2020). High stromal nicotinamide N-methyltransferase (NNMT) indicates poor prognosis in colorectal cancer. Cancer Med..

[47] Rogers C.D., Fukushima N., Sato N., Shi C., Prasad N., Hustinx S.R., Matsubayashi H., Canto M., Eshleman J.R., Hruban R.H. (2006). Differentiating pancreatic lesions by microarray and QPCR analysis of pancreatic juice RNAs. Cancer Biol. Ther..

[48] Yu T., Wang Y.T., Chen P., Li Y.H., Chen Y.X., Zeng H., Yu A.M., Huang M., Bi H.C. (2015). Effects of nicotinamide N-methyltransferase on PANC-1 cells proliferation, metastatic potential and survival under metabolic stress. Cell Physiol. Biochem..

[49] Tomida M., Mikami I., Takeuchi S., Nishimura H., Akiyama H. (2009). Serum levels of nicotinamide N-methyltransferase in patients with lung cancer. J. Cancer Res. Clin. Oncol..

[50] Sartini D., Morganti S., Guidi E., Rubini C., Zizzi A., Giuliante R., Pozzi V., Emanuelli M. (2013). Nicotinamide N-methyltransferase in non-small cell lung cancer: Promising results for targeted anti-cancer therapy. Cell Biochem. Biophys..

[51] Sartini D., Seta R., Pozzi V., Morganti S., Rubini C., Zizzi A., Tomasetti M., Santarelli L., Emanuelli M. (2015). Role of nicotinamide N-methyltransferase in non-small cell lung cancer: In vitro effect of shRNA-mediated gene silencing on tumorigenicity. Biol. Chem..

[52] Bach D.H., Kim D., Bae S.Y., Kim W.K., Hong J.Y., Lee H.J., Rajasekaran N., Kwon S., Fan Y., Luu T.T. (2018). Targeting Nicotinamide N-Methyltransferase and miR-449a in EGFR-TKI-Resistant Non-Small-Cell Lung Cancer Cells. Mol. Ther. Nucleic Acids.

[53] Sartini D., Santarelli A., Rossi V., Goteri G., Rubini C., Ciavarella D., Lo Muzio L., Emanuelli M. (2007). Nicotinamide N-methyltransferase upregulation inversely correlates with lymph node metastasis in oral squamous cell carcinoma. Mol. Med..

[54] Emanuelli M., Santarelli A., Sartini D., Ciavarella D., Rossi V., Pozzi V., Rubini C., Lo Muzio L. (2010). Nicotinamide N-methyltransferase upregulation correlates with tumour differentiation in oral squamous cell carcinoma. Histol. Histopathol..

[55] Pozzi V., Mazzotta M., Lo Muzio L., Sartini D., Santarelli A., Renzi E., Rocchetti R., Tomasetti M., Ciavarella D., Emanuelli M. (2011). Inhibiting proliferation in KB cancer cells by RNA interference-mediated knockdown of nicotinamide N-methyltransferase expression. Int. J. Immunopathol. Pharmacol..

[56] Sartini D., Pozzi V., Renzi E., Morganti S., Rocchetti R., Rubini C., Santarelli A., Lo Muzio L., Emanuelli M. (2012). Analysis of tissue and salivary nicotinamide N-methyltransferase in oral squamous cell carcinoma: Basis for the development of a noninvasive diagnostic test for early-stage disease. Biol. Chem..

[57] Pozzi V., Sartini D., Morganti S., Giuliante R., Di Ruscio G., Santarelli A., Rocchetti R., Rubini C., Tomasetti M., Giannatempo G. (2013). RNA-mediated gene silencing of nicotinamide N-methyltransferase is associated with decreased tumorigenicity in human oral carcinoma cells. PLoS ONE.

[58] Ishibashi K., Ishii K., Sugiyama G., Sumida T., Sugiura T., Kamata Y.U., Seki K., Fujinaga T., Kumamaru W., Kobayashi Y. (2018). Deregulation of Nicotinamide N-Methyltransferase and Gap Junction Protein Alpha-1 Causes Metastasis in Adenoid Cystic Carcinoma. Anticancer Res..

[59] Seta R., Mascitti M., Campagna R., Sartini D., Fumarola S., Santarelli A., Giuliani M., Cecati M., Lo Muzio L., Emanuelli M. (2019). Overexpression of Nicotinamide N-Methyltransferase in HSC-2 OSCC cell line: Effect on apoptosis and cell proliferation. Clin. Oral. Investig..

[60] Cui Y., Zhang L., Wang W., Ma S., Liu H., Zang X., Zhang Y., Guan F. (2019). Downregulation of nicotinamide N-methyltransferase inhibits migration and epithelial-mesenchymal transition of esophageal squamous cell carcinoma via Wnt/beta-catenin pathway. Mol. Cell. Biochem..

[61] Cui Y., Yang D., Wang W., Zhang L., Liu H., Ma S., Guo W., Yao M., Zhang K., Li W. (2020). Nicotinamide N-methyltransferase decreases 5-fluorouracil sensitivity in human esophageal squamous cell carcinoma through metabolic reprogramming and promoting the Warburg effect. Mol. Carcinog..

[62] Win K.T., Lee S.W., Huang H.Y., Lin L.C., Lin C.Y., Hsing C.H., Chen L.T., Li C.F. (2013). Nicotinamide N-methyltransferase overexpression is associated with Akt phosphorylation and indicates worse prognosis in patients with nasopharyngeal carcinoma. Tumour Biol..

[63] Xu J., Moatamed F., Caldwell J.S., Walker J.R., Kraiem Z., Taki K., Brent G.A., Hershman J.M. (2003). Enhanced expression of nicotinamide N-methyltransferase in human papillary thyroid carcinoma cells. J. Clin. Endocrinol. Metab..

[64] Xu J., Capezzone M., Xu X., Hershman J.M. (2005). Activation of nicotinamide N-methyltransferase gene promoter by hepatocyte nuclear factor-1beta in human papillary thyroid cancer cells. Mol. Endocrinol..

[65] Xu J., Hershman J.M. (2006). Histone deacetylase inhibitor depsipeptide represses nicotinamide N-methyltransferase and hepatocyte nuclear factor-1beta gene expression in human papillary thyroid cancer cells. Thyroid.

[66] Mascitti M., Sartini D., Togni L., Pozzi V., Rubini C., Santarelli A., Emanuelli M. (2020). Differential expression of nicotinamide N-methyltransferase in primary and recurrent ameloblastomas and odontogenic keratocysts. Eur. J. Clin. Invest..

[67] Markert J.M., Fuller C.M., Gillespie G.Y., Bubien J.K., McLean L.A., Hong R.L., Lee K., Gullans S.R., Mapstone T.B., Benos D.J. (2001). Differential gene expression profiling in human brain tumors. Physiol. Genomics.

[68] Ganzetti G., Sartini D., Campanati A., Rubini C., Molinelli E., Brisigotti V., Cecati M., Pozzi V., Campagna R., Offidani A. (2018). Nicotinamide N-methyltransferase: Potential involvement in cutaneous malignant melanoma. Melanoma Res..

[69] Mascitti M., Santarelli A., Sartini D., Rubini C., Colella G., Salvolini E., Ganzetti G., Offidani A., Emanuelli M. (2019). Analysis of nicotinamide N-methyltransferase in oral malignant melanoma and potential prognostic significance. Melanoma Res..

[70] Pompei V., Salvolini E., Rubini C., Lucarini G., Molinelli E., Brisigotti V., Pozzi V., Sartini D., Campanati A., Offidani A. (2019). Nicotinamide N-methyltransferase in nonmelanoma skin cancers. Eur. J. Clin. Investig..

[71] Hah Y.S., Cho H.Y., Jo S.Y., Park Y.S., Heo E.P., Yoon T.J. (2019). Nicotinamide N-methyltransferase induces the proliferation and invasion of squamous cell carcinoma cells. Oncol. Rep..

[72] Sartini D., Pompei V., Lucarini G., Rubini C., Molinelli E., Brisigotti V., Salvolini E., Campanati A., Offidani A., Emanuelli M. (2020). Differential expression of nicotinamide n-methyltransferase in cutaneous keratoacanthoma and squamous cell carcinoma: An immunohistochemical study. J. Eur. Acad. Dermatol. Venereol..

[73] Campagna R., Salvolini E., Pompei V., Pozzi V., Salvucci A., Molinelli E., Brisigotti V., Sartini D., Campanati A., Offidani A. (2021). Nicotinamide N-Methyltransferase Gene Silencing Enhances Chemosensitivity of Melanoma Cell Lines. Pigment Cell Melanoma Res..

[74] D’Andrea F.P., Safwat A., Kassem M., Gautier L., Overgaard J., Horsman M.R. (2011). Cancer stem cell overexpression of nicotinamide N-methyltransferase enhances cellular radiation resistance. Radiother. Oncol..

[75] Ulanovskaya O.A., Zuhl A.M., Cravatt B.F. (2013). NNMT promotes epigenetic remodeling in cancer by creating a metabolic methylation sink. Nat. Chem. Biol..

[76] Pozzi V., Sartini D., Rocchetti R., Santarelli A., Rubini C., Morganti S., Giuliante R., Calabrese S., Di Ruscio G., Orlando F. (2015). Identification and Characterization of Cancer Stem Cells from Head and Neck Squamous Cell Carcinoma Cell Lines. Cell. Physiol. Biochem..

[77] Jung J., Kim L.J., Wang X., Wu Q., Sanvoranart T., Hubert C.G., Prager B.C., Wallace L.C., Jin X., Mack S.C. (2017). Nicotinamide metabolism regulates glioblastoma stem cell maintenance. JCI Insight.

[78] Pozzi V., Salvolini E., Lucarini G., Salvucci A., Campagna R., Rubini C., Sartini D., Emanuelli M. (2020). Cancer stem cell enrichment is associated with enhancement of nicotinamide N-methyltransferase expression. IUBMB Life.

[79] Zhang J., Wang Y., Li G., Yu H., Xie X. (2014). Down-regulation of nicotinamide N-methyltransferase induces apoptosis in human breast cancer cells via the mitochondria-mediated pathway. PLoS ONE.

[80] Wang Y., Zeng J., Wu W., Xie S., Yu H., Li G., Zhu T., Li F., Lu J., Wang G.Y. (2019). Nicotinamide N-methyltransferase enhances chemoresistance in breast cancer through SIRT1 protein stabilization. Breast Cancer Res..

[81] Yu H., Zhou X., Wang Y., Huang X., Yang J., Zeng J., Li G., Xie X., Zhang J. (2020). Nicotinamide N-methyltransferase inhibits autophagy induced by oxidative stress through suppressing the AMPK pathway in breast cancer cells. Cancer Cell Int..

[82] Akar S., Harmankaya İ., Uğraş S., Çelik Ç. (2019). Nicotinamide N-methyltransferase expression and its association with phospho-Akt, p53 expression, and survival in high-grade endometrial cancer. Turk. J. Med. Sci..

[83] Akar S., Harmankaya İ., Uğraş S., Çelik Ç. (2020). Expression and Clinical Significance of Nicotinamide N-Methyltransferase in Cervical Squamous Cell Carcinoma. Int. J. Gynecol. Pathol..

[84] Harmankaya İ., Akar S., Uğraş S., Güler A.H., Ezveci H., Aydoğdu M., Çelik Ç. (2020). Nicotinamide N-methyltransferase overexpression may be associated with poor prognosis in ovarian cancer. J. Obstet. Gynaecol..

[85] Ferlay J., Soerjomataram I., Dikshit R., Eser S., Mathers C., Rebelo M., Parkin D.M., Forman D., Bray F. (2015). Cancer incidence and mortality worldwide: Sources, methods and major patterns in GLOBOCAN 2012. Int. J. Cancer..

[86] Shuch B., Amin A., Armstrong A.J., Eble J.N., Ficarra V., Lopez-Beltran A., Martignoni G., Rini B.I., Kutikov A. (2015). Understanding pathologic variants of renal cell carcinoma: Distilling therapeutic opportunities from biologic complexity. Eur. Urol..

[87] Capitanio U., Montorsi F. (2016). Renal cancer. Lancet..

[88] Hancock S.B., Georgiades C.S. (2016). Kidney Cancer. Cancer J..

[89] Yao M., Tabuchi H., Nagashima Y., Baba M., Nakaigawa N., Ishiguro H., Hamada K., Inayama Y., Kishida T., Hattori K. (2005). Gene expression analysis of renal carcinoma: Adipose differentiation-related protein as a potential diagnostic and prognostic biomarker for clear-cell renal carcinoma. J. Pathol..

[90] Sartini D., Muzzonigro G., Milanese G., Pierella F., Rossi V., Emanuelli M. (2006). Identification of nicotinamide N-methyltransferase as a novel tumor marker for renal clear cell carcinoma. J. Urol..

[91] Zhang J., Xie X.Y., Yang S.W., Wang J., He C. (2010). Nicotinamide N-methyltransferase protein expression in renal cell cancer. J. Zhejiang Univ. Sci. B.

[92] Kim D.S., Choi Y.P., Kang S., Gao M.Q., Kim B., Park H.R., Choi Y.D., Lim J.B., Na H.J., Kim H.K. (2010). Panel of candidate biomarkers for renal cell carcinoma. J. Proteome Res..

[93] Su Kim D., Choi Y.D., Moon M., Kang S., Lim J.B., Kim K.M., Park K.M., Cho N.H. (2013). Composite three-marker assay for early detection of kidney cancer. Cancer Epidemiol. Biomark. Prev..

[94] Kim D.S., Ham W.S., Jang W.S., Cho K.S., Choi Y.D., Kang S., Kim B., Kim K.J., Lim E.J., Rha S.Y. (2020). Scale-Up Evaluation of a Composite Tumor Marker Assay for the Early Detection of Renal Cell Carcinoma. Diagnostics.

[95] Neely B.A., Wilkins C.E., Marlow L.A., Malyarenko D., Kim Y., Ignatchenko A., Sasinowska H., Sasinowski M., Nyalwidhe J.O., Kislinger T. (2016). Proteotranscriptomic Analysis Reveals Stage Specific Changes in the Molecular Landscape of Clear-Cell Renal Cell Carcinoma. PLoS ONE.

[96] Song Y., Zhong L., Zhou J., Lu M., Xing T., Ma L., Shen J. (2017). Data-Independent Acquisition-Based Quantitative Proteomic Analysis Reveals Potential Biomarkers of Kidney Cancer. Proteom. Clin. Appl..

[97] Campagna R., Cecati M., Pozzi V., Fumarola S., Pompei V., Milanese G., Galosi A.B., Sartini D., Emanuelli M. (2018). Involvement of transforming growth factor beta 1 in the transcriptional regulation of nicotinamide N-methyltransferase in clear cell renal cell carcinoma. Cell. Mol. Biol..

[98] Tang S.W., Yang T.C., Lin W.C., Chang W.H., Wang C.C., Lai M.K., Lin J.Y. (2011). Nicotinamide N-methyltransferase induces cellular invasion through activating matrix metalloproteinase-2 expression in clear cell renal cell carcinoma cells. Carcinogenesis.

[99] Kwon Y., Song J., Lee H., Kim E.Y., Lee K., Lee S.K., Kim S. (2015). Design, Synthesis, and Biological Activity of Sulfonamide Analogues of Antofine and Cryptopleurine as Potent and Orally Active Antitumor Agents. J. Med. Chem..

[100] Sartini D., Muzzonigro G., Milanese G., Pozzi V., Vici A., Morganti S., Rossi V., Mazzucchelli R., Montironi R., Emanuelli M. (2013). Upregulation of tissue and urinary nicotinamide N-methyltransferase in bladder cancer: Potential for the development of a urine-based diagnostic test. Cell Biochem. Biophys..

[101] Riester M., Taylor J.M., Feifer A., Koppie T., Rosenberg J.E., Downey R.J., Bochner B.H., Michor F. (2012). Combination of a novel gene expression signature with a clinical nomogram improves the prediction of survival in high-risk bladder cancer. Clin. Cancer Res..

[102] Kirkali Z., Chan T., Manoharan M., Algaba F., Busch C., Cheng L., Kiemeney L., Kriegmair M., Montironi R., Murphy W.M. (2005). Bladder cancer: Epidemiology, staging and grading, and diagnosis. Urology.

[103] Kim W.J., Kim S.K., Jeong P., Yun S.J., Cho I.C., Kim I.Y., Moon S.K., Um H.D., Choi Y.H. (2011). A four-gene signature predicts disease progression in muscle invasive bladder cancer. Mol. Med..

[104] Kim W.J., Bae S.C. (2008). Molecular biomarkers in urothelial bladder cancer. Cancer Sci..

[105] Guidance N.I. (2017). Bladder cancer: Diagnosis and management of bladder cancer. BJU Int..

[106] Pozzi V., Di Ruscio G., Sartini D., Campagna R., Seta R., Fulvi P., Vici A., Milanese G., Brandoni G., Galosi A.B. (2018). Clinical performance and utility of a NNMT-based urine test for bladder cancer. Int. J. Biol. Mark..

[107] Kassem H.S., Sangar V., Cowan R., Clarke N., Margison G.P. (2002). A potential role of heat shock proteins and nicotinamide N-methyl transferase in predicting response to radiation in bladder cancer. Int. J. Cancer.

[108] Wu Y., Siadaty M.S., Berens M.E., Hampton G.M., Theodorescu D. (2008). Overlapping gene expression profiles of cell migration and tumor invasion in human bladder cancer identify metallothionein 1E and nicotinamide N-methyltransferase as novel regulators of cell migration. Oncogene.

[109] Nguyen-Nielsen M., Borre M. (2016). Diagnostic and Therapeutic Strategies for Prostate Cancer. Semin. Nucl. Med..

[110] Misawa A., Takayama K.I., Inoue S. (2017). Long non-coding RNAs and prostate cancer. Cancer Sci..

[111] Zhou W., Gui M., Zhu M., Long Z., Huang L., Zhou J., He L., Zhong K. (2014). Nicotinamide N-methyltransferase is overexpressed in prostate cancer and correlates with prolonged progression-free and overall survival times. Oncol. Lett..

[112] You Z., Liu Y., Liu X. (2018). Nicotinamide N-methyltransferase enhances the progression of prostate cancer by stabilizing sirtuin 1. Oncol. Lett..

[113] Campagna R., Mateuszuk Ł., Wojnar-Lason K., Kaczara P., Tworzydło A., Kij A., Bujok R., Mlynarski J., Wang Y., Sartini D. (2021). Nicotinamide N-methyltransferase in endothelium protects against oxidant stress-induced endothelial injury. Biochim. Biophys. Acta Mol. Cell. Res..

[114] Pissios P. (2017). Nicotinamide N-Methyltransferase: More Than a Vitamin B3 Clearance Enzyme. Trends Endocrinol. Metab..

[115] Roberti A., Fernández A.F., Fraga M.F. (2021). Nicotinamide N-methyltransferase: At the crossroads between cellular metabolism and epigenetic regulation. Mol. Metab..

[116] Novak Kujundžić R., Prpić M., Đaković N., Dabelić N., Tomljanović M., Mojzeš A., Fröbe A., Trošelj K.G. (2021). Nicotinamide N-Methyltransferase in Acquisition of Stem Cell Properties and Therapy Resistance in Cancer. Int. J. Mol. Sci..

